# High progesterone levels in women with high ovarian response do not affect clinical outcomes: a retrospective cohort study

**DOI:** 10.1186/1477-7827-12-69

**Published:** 2014-07-26

**Authors:** Antonio Requena, María Cruz, Ernesto Bosch, Marcos Meseguer, Juan Antonio García-Velasco

**Affiliations:** 1 Reproductive Medicine Department, Instituto Valenciano de Infertilidad IVI Madrid, Avenida del Talgo 68-70, Aravaca, Madrid 28023, Spain; 2 Reproductive Medicine Department, Instituto Valenciano de Infertilidad IVI Valencia, Plaza de la Policía Local 3, Valencia 46015, Spain; 3 Nursing, Gynecology and Obstetrics, Pediatrics and Psychiatry Department, Faculty of Health Sciences, Rey Juan Carlos University, Avda. Atenas s/n, Alcorcón, Madrid 28922, Spain

**Keywords:** Ovarian response, High responders, High progesterone, Clinical outcomes

## Abstract

**Background:**

The potentially detrimental role of progesterone during the follicular phase has been a matter of controversy for several years; however, few studies have analyzed the effects of combined raised estradiol and progesterone levels on pregnancy outcomes. The aim of the present study was to determine the influence of high progesterone levels on clinical outcomes in the context of high ovarian response.

**Methods:**

We performed a retrospective cohort study that included 2850 women classified as high responders. The women were subdivided into six groups depending on their progesterone concentration on the day of human chorionic gonadotropin (hCG) administration: <0.5 ng/ml (<p10), 0.50-0.70 ng/ml (p10-p25), 0.71-1.00 ng/ml (p25-p50), 1.01-1.40 ng/ml (p50-p75), 1.41-1.80 ng/ml (p75-p90) and >1.81 ng/ml (>p90). Ovarian response was classified as high when > =20 oocytes were retrieved or when estradiol was > =3000 pg/ml. Clinical outcomes of each subgroup were analyzed. We also examined data from frozen-thawed embryo transfers. Results were analyzed with Student’s t- test to compare continuous variables and chi-squared test to compare proportions. A *p*-value of < =0.05 was considered statistically significant.

**Results:**

The progesterone concentration increased with ovarian response, and elevated progesterone did not show a significant clinical impact on implantation rate and pregnancy rates. These data provide evidence that progesterone levels higher than 1.8 ng/ml do not have detrimental effect on oocyte quality or endometrial receptivity.

**Conclusions:**

These data allow us to conclude that high progesterone levels correlate significantly with high estradiol levels and that in high responder women; progesterone levels do not show a significant clinical impact on results.

## Background

Control over estradiol secretion is explained by the two cell- two gonadotropins theory, while control of progesterone synthesis is less well understood. Progesterone plays a key role during the second phase of the menstrual cycle and is particularly important for implantation and progression of pregnancy; however, in the context of stimulated cycles, the potentially detrimental role of this hormone during the follicular phase has been the focus of interest and a matter of controversy for several years. Increased progesterone levels during the early follicular phase in women undergoing controlled ovarian stimulation have been suggested to be associated with a decreased probability of ongoing pregnancy [1-3]; however, many other studies have found no association between these two parameters [4,5]. The pathogenesis of raised peripheral concentrations of progesterone in the late follicular phase is likely to influence the secretory changes of the endometrium, leading to impaired endometrial receptivity [6]. On the other hand, patients who respond adequately to controlled ovarian stimulation are more likely to produce better oocytes. Therefore, the consequences of elevated progesterone levels on clinical outcomes in patients with good response are likely to result from a balance between two antagonistic factors: the probability of getting more oocytes associated with a good ovarian response and the impaired receptivity of the endometrium resulting from the premature endometrial exposure to progesterone [7].

Although increased progesterone levels have historically been associated with LH in the context of premature LH surges, some studies have indicated that the elevated progesterone is due to FSH exposure during the follicular phase [8,9]. Changes in paracrine regulation might explain the increase in progesterone production observed in the late follicular phase [10]; this is a pathological mechanism that is probably associated with the high response of the ovary during stimulation, which results in an excessive number of follicles and proliferating granulose cells. It was demonstrated recently that patients with high estradiol concentrations have significantly higher progesterone concentrations [11,12]; thus, this association suggests that at least one of the mechanisms that play a role in increased progesterone levels is linked to a high ovarian response during controlled ovarian stimulation. This idea has been recently confirmed by Griesinger et al. [13] which show that the incidence of progesterone elevation on the day of hCG increased with the ovarian response from 4.5% in low responders to 19.0% in high responders.

Changes in hormone concentrations convey significant biological messages; understanding and proving this theory could give rise to better approaches to treatment and risk assessment. Most studies of the association between steroid hormones and clinical outcomes were limited to investigating elevated levels of progesterone or estradiol individually, rather than in combination. Few studies have analyzed the effects of combined raised estradiol and progesterone levels on pregnancy outcomes. Therefore, the aim of the present study was to determine the influence of high progesterone levels on clinical outcomes in the context of high ovarian response.

## Methods

### Study population and design

This was a non-interventional, large-sample, retrospective, multicenter cohort study of patients who underwent routine clinical examinations and procedures. A total of 2850 infertile women who were classified as high responders and were undergoing assisted reproduction techniques from January 2009 to December 2011 were included in this retrospective study. These women received treatment at one of 11 private clinics belonging to the IVI Group in Spain. To reflect the broad range of patients typically encountered in clinical practice, no inclusion/exclusion criteria were applied regarding baseline characteristics. High response was defined as women who had ≥20 oocytes retrieved or whose estradiol levels were ≥3000 pg/ml.

All patients signed written informed consent forms. All procedures and protocols were approved by an Institutional Review Board (reference MAD-AR-09-2013-01). It complied with the Spanish law governing assisted reproduction technologies (14/2006).

Patients underwent controlled ovarian stimulation with a short GnRH antagonist protocol triggered with human chorionic gonadotropin (hCG) for pituitary down-regulation. Ovarian stimulation was performed by one of four possible methods: recombinant follicle stimulating hormone (rFSH) alone (Gonal-F, Merck-Serono, Spain; or Puregon, MSD, Spain); rFSH combined with recombinant luteinizing hormone (rLH) (Luveris, Merck-Serono, Spain); highly purified human menopausal gonadotropin (HP-hMG) alone (Menopur, Ferring Pharmaceutical, Spain); or rFSH combined with HP-hMG. Because this was a retrospective study, no specific criteria were defined for the selection of gonadotropins; choices were made on a case-by-case basis according to patient characteristics and clinician preferences. The initial dose of gonadotropin was individualized for each patient according to age, basal FSH levels, antral follicle count, body mass index (BMI), and previous response to ovarian stimulation. Dose adjustments were performed according to ovarian response, which was monitored by vaginal scans and estradiol determinations.

After oocyte retrieval, in *vitro* fertilization (IVF) or intracytoplasmic sperm injection (ICSI) was performed. The quality of all available embryos was evaluated, and up to two embryos were transferred on day 3 of development. Evaluated parameters on developmental day 3 included cell number, symmetry, granularity, type and percentage of fragmentation, presence of multinucleate blastomeres, and degree of compaction as previously described [14]. A top-quality embryo was described as 4–5 cells on day 2, > = 7 cells on day 3, equally sized blastomeres and < =20% fragmentation on day 3, and no multinucleate cells.

The β-hCG concentration was determined 13 days after embryo transfer, and the clinical pregnancy and implantation rate was confirmed when a gestational sac with fetal heart beat was visible by ultrasound examination after 7 weeks of pregnancy.

The primary objective was to determine the relationship between serum progesterone levels on the day of hCG administration and the implantation and pregnancy rates in high responder patients. As a secondary objective, we also tried to establish, when appropriate, a serum progesterone threshold in this specific set of patients that would define circulating progesterone levels that were detrimental to cycle outcomes.

### Hormonal measurements

Blood samples for the analysis of circulating estradiol and progesterone were assessed on the day of hCG administration. Serum samples were analyzed by chemiluminescence with the Architect analyzer (Abbot Diagnostics, Spain). The analytical sensitivity of the estradiol assay was ≤10 pg/ml, with a coefficient of variation of ≤7%. The analytical sensitivity of the progesterone assay was ≤0.1 ng/ml, with a coefficient of variation of ≤7%.

### Statistical analysis

To describe the distribution of the probabilities of implantation and pregnancy rates, progesterone concentrations were converted from continuous variables into categorical variables by dividing them into groups based on their percentiles. By employing this procedure, we avoided bias by assuming that any relationship between serum progesterone level and clinical outcome may be linear. Next, patients were separated into percentiles (p10, p25, p50, p75 and p90) and then subdivided into six distinct groups according to their serum progesterone levels on the day of hCG administration: <0.5 ng/ml (<p10, *n* = 228), 0.50-0.70 ng/ml (p10-p25, *n* = 409), 0.71-1.00 ng/ml (p25-p50, *n* = 738), 1.01-1.40 ng/ml (p50-p75, *n* = 752), 1.41-1.80 ng/ml (p75-p90, *n* = 381) and >1.81 ng/ml (>p90, *n* = 342).

The odds ratio (OR) of all of the variables generated on implantation and pregnancy was expressed in terms of 95% confidence interval (95% CI); the effect of progesterone was quantified by conducting a logistic regression analysis. The receiver operating characteristic (ROC) curve was employed to test the predictive value of the variable progesterone levels included in the model with respect to implantation in high ovarian response patients. ROC curve analysis provides values for the area under the curve (AUC) that are between 0.5 and 1.0 and can be interpreted as a measurement of the global classification ability of the model.

Finally, values are expressed as mean ± standard deviation (SD). One-way analysis of variance (ANOVA) with Bonferroni post-hoc tests for continuous variables and the chi-squared test for categorical data were used for data analysis. Statistical analysis was performed with the Statistical Package for Social Sciences version 19.0 (SPSS, Chicago, IL, USA), and *p* < 0.05 was considered statistically significant.

## Results

### Overall results

A total of 2850 women defined as high responders and undergoing GnRH antagonist cycles were included in this study. We performed IVF or ICSI. Clinical characteristics of the study population are presented in Table 1. Fresh embryo transfer was performed in 1990 cycles (69.8%), while 860 patients (30.2%) froze all of their embryos and underwent a later frozen-thawed embryo transfer (FET).

**Table 1:** Clinical characteristics of the study population

**Parameters**	**Values**
**Age (years)**	34.8 (19–42)
**BMI (kg/m2)**	23.0 (19.2-31.4)
**Estradiol (pg/ml)**	3578.8 ± 1232
**Progesterone (ng/ml)**	1.3 ± 0.6
**N of oocytes retrieved**	19.6 (0–32)
**N transferred embryos**	1.75 (0–3)
**N frozen embryos**	5.6 (0–12)

### High progesterone levels in high ovarian response

**Figure 1:**
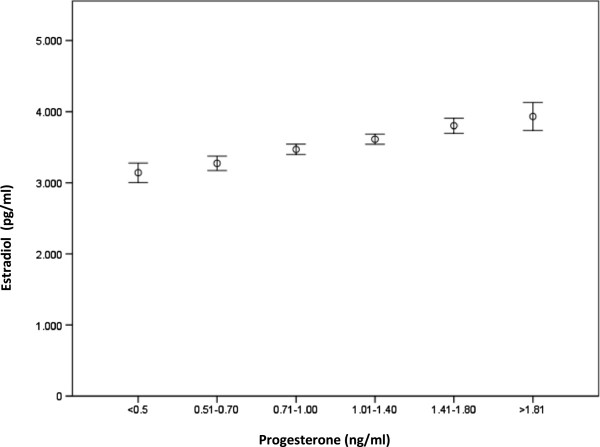
High estradiol concentrations are associated with high estradiol concentrations.

Estradiol concentrations were significantly higher as long as the progesterone concentration increased, (*p* < 0.001, Figure 1).

We observed no significant differences (*p* = 0.456) in the mean progesterone concentration with respect to the type of gonadotropin that was used for ovarian stimulation: rFSH alone (*n* = 728, progesterone 1.06 ng/ml), rFSH + rLH (*n* = 377, progesterone 1.01 ng/ml), HP-hMG alone (*n* = 370; progesterone 1.10 ng/ml); and rFSH + HP-hMG (*n* = 1375; progesterone 1.30 ng/ml).

**Figure 2:**
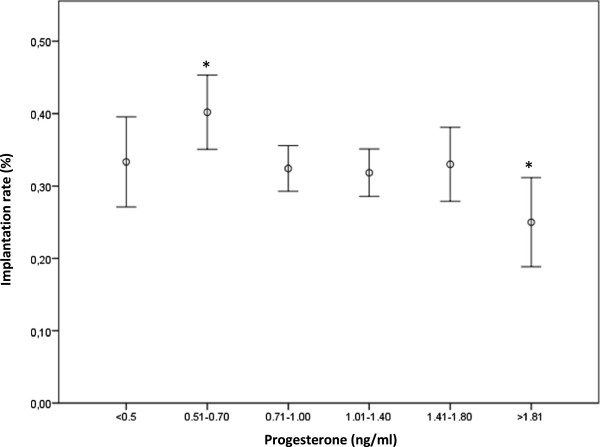
**Implantation rates according progesterone levels.** (*, p = 0.045).

In fresh cycles, we observed a marginal significant difference (*p =* 0.045) in implantation rates between patients within the progesterone interval 0.51-0.70 ng/ml (p10-p25) and patients with progesterone levels ≥1.80 ng/ml (>p90) (Figure 2); the OR associated with the effect of progesterone on the implantation rate was -0.056 (95% CI 0.031 to -0.004). These data suggest that a serum progesterone concentration of >1.8 ng/ml may represent the threshold level above which we can begin to observe some kind of effect on the implantation rate; however, the AUC was ≤ 0.5 (AUC = 0.486), showing that the analysis was not informative.

**Figure 3:**
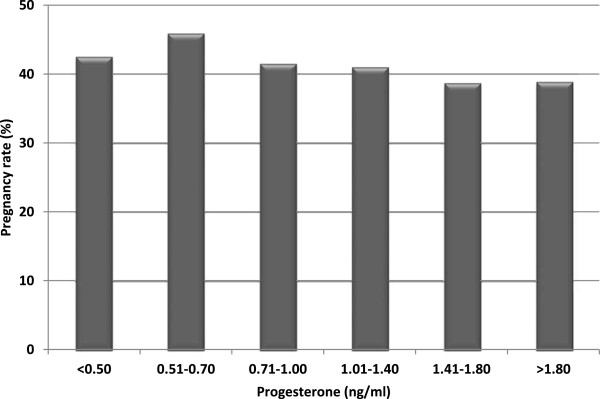
**Pregnancy rates according progesterone levels.** (*, p = 0.044).

Regarding pregnancy rates in fresh cycles, we also observed minor significant differences (*p* = 0.044, Figure 3) in patients with progesterone levels >1.8 ng/ml; moreover, when we re-analyzed the data considering only two groups (<p90 and > p90), we observed that the pregnancy rate decreased (*p* = 0.048) when progesterone levels were >1.8 ng/ml (>p90) compared to patients with progesterone levels below this value (57.1% vs. 50.9%, respectively). The OR associated with this probability was 0.73 (95% CI 0.61 to 0.99). As well as we checked for the implantation rate, the ROC curve (AUC = 0.455) failed to find any prediction about the influence of high progesterone levels on pregnancy rates within a context of high ovarian response.

Among all of the patients with high ovarian response, 860 underwent a FET cycle instead of fresh embryo transfer, because all of their embryos were frozen. Mean serum progesterone concentration in FET cycles was 1.6 ± 0.8 ng/ml. We did not observe any significant differences in the implantation rate (*p* = 0.529) among the progesterone subgroups: <p10, 36.7%; p10-25, 38.1%; p25-p50, 39.6%; p50-p75, 32.9%; p75-p90, 33.1%; and > p90, 38.8%. We obtained similar results regarding the pregnancy rate: <p10, 68.0%; p10-p25, 60.0%; p25-p50, 69.2%; p50-p75, 57.0%; p75-p90, 56.2%; and > p90, 58.3% (*p* = 0.166).

## Discussion

Supraphysiological serum estradiol levels are closely related to the ovarian stimulation that is necessary for multiple follicle development. Based on the fact that ovarian steroid hormones regulate cyclic changes in the endometrium, increased estradiol levels during controlled ovarian stimulation may compromise endometrial receptivity for embryo implantation. Several authors have suggested that high responders have significantly lower implantation and pregnancy rates in addition to impaired endometrial receptivity [15], while others have failed to find an association between high estradiol levels on the day of hCG administration and harmful effect on pregnancy outcomes [16,17]. Although progesterone was not analyzed in those studies, other researchers have proposed that not only estradiol levels, but also progesterone concentrations, affect pregnancy outcomes [1,18]. Nevertheless, the unfavorable effect of elevated progesterone on pregnancy outcomes has been questioned in other reviews [4,19,20].

While a wide range of estradiol concentrations during the late follicular phase are able to accommodate successful pregnancies [11,21,22], there is somewhat more controversy regarding progesterone concentrations in the late follicular phase. When we analyzed the data with respect to fresh cycles, the results of the present retrospective study showed that in women classified as high responders, implantation rates were barely significantly different between women with 0.5-0.7 ng/ml (p10-p25) and the highest progesterone intervals. Our data suggest that a serum progesterone concentration exceeding 1.8 ng/ml may represent the value at which progesterone begins to have a minimum effect on implantation rates in patients with high ovarian response. However, the AUC derived from ROC analysis exhibited an uninformative value, which suggests that progesterone concentration in high responders is not a determinant factor that can be used to predict clinical outcomes. These results remained the same with respect to pregnancy rates; in both cases, the cut-off point beyond which high progesterone could affect clinical data shifted to higher concentrations than those observed in women with normal ovarian response. These results are in agreement with previous reports [13] that showed that the incidence of elevated progesterone increases with ovarian response and that elevated progesterone is not associated with a decreased chance of pregnancy in high responders. These results have important connotations because clinical decisions, especially those related with freezing all available embryos for transferring them in a subsequent cycle, are a common issue in cycles with high progesterone levels the day of triggering. According to these data it might be possible to offer a delayed transfer as a way to avoid the risk of OHSS and not when we find high progesterone levels at late follicular phase, as they seem not to affect pregnancy rates. In summary, we showed that there was a slight but significant association between serum progesterone values in the late follicular phase and clinical outcomes, although this significance may have little impact at clinical level.

The decreasing trend between progesterone concentration and clinical outcomes is in agreement with the results previously reported by Bosch et al. [3], although our results did not reveal such a large difference in outcomes among various progesterone concentrations. The minimal impact of progesterone levels in high responders on the pregnancy rate suggests that the negative effect predicted for elevated progesterone could be overcome by other factors with a positive effect; it is possible that high responders may have better and faster developing embryos that “catch up” with the hypothetical endometrial changes owing to a premature increase in progesterone [21]. Moreover, and in agreement with this hypothesis, it has been reported that embryos derived from women with progesterone levels >1.1 ng/ml cleaved significantly faster [23], which supports the idea that these embryos circumvent the endometrial changes associated with progesterone secretion.

In addition, high responders exhibited a closer relationship between estradiol and progesterone levels, which is in agreement with a theory that suggests that at least one of the mechanisms that play a role in the premature increase of plasma progesterone is linked to the high ovarian response of the ovary to controlled ovarian stimulation [11]. This hypothesis (and consequently our data) has been confirmed through a logistic regression model, which established that the number of follicles on the day of hCG administration might predict serum progesterone levels [12]. What is more, the paper from Griesinger [13] and our own results showed that women with high progesterone were generally high responders; these findings confirm that each individual follicle contributed to the collective observed concentration and that high serum estradiol levels and number of oocyte retrieved were associated with high progesterone concentrations.

Progesterone elevation may be partially caused by a lack of uniform stimulation, as different forms containing LH were included. Although we did not account for LH activity, we analyzed progesterone concentrations according to the type of gonadotropin used during controlled ovarian stimulation, and we did not observe significant differences among them. Moreover, the absence of significant differences between the different types of gonadotropin illustrates that neither FSH-like nor LH-like activity each have an effect on follicular progesterone synthesis, and that serum progesterone levels in high responders are determined by the degree of ovarian response instead of the type of gonadotropin. These data do not reconcile with previous reports that found significant differences in progesterone levels when compared recombinant FSH vs. hMG [10]; these differences remained even after adjusting the results for ovarian response. In this later paper, the number of women considered as high ovarian responders was too small (n = 30 in the recombinant FSH group and n = 35 in the hMG group), then these quantities contrast sharply with our sample size, which might explain why we did not find significant differences in progesterone concentrations when compared different types of medication.

It has also been suggested that raised progesterone concentrations are likely to influence endometrial development, which could in turn lead to a change in the normal synchrony between the embryo and the endometrium when excessively disturbed [24-26]. One strategy to prevent the hypothetical effects of progesterone elevation on uterine receptivity might be to vitrify all embryos and then transfer embryos during a later cycle [27]. It is worthwhile to remember the retrospective character of this study and the fact that data collection ended in 2011; at that time, vitrification and the use of a GnRH agonist to induce ovulation were not systematically implemented in daily clinical practice, which explains the proportion of fresh embryo transfers performed in high ovarian responders. When we analyzed clinical outcomes in FET cycles, we did not observe significant differences in any of the variables. These findings confirm that progesterone elevation does not have a negative effect at the level of embryo quality [28,29] but it does have a slight influence on endometrial development which could be overwhelmed by the embryo itself in this specific context of high ovarian response. At this point, since some clinicians have recommended that all embryos be frozen and transferred during a later treatment, it might seem appealing that cycles could be managed by fresh embryo transfers in high responders.

## Conclusions

Our results confirmed that the risk of a pre-ovulatory rise in progesterone levels increases with the ovarian response. In addition, a direct comparison of clinical outcomes among women with high ovarian response who were classified in different progesterone concentration intervals revealed a gradually decreasing trend when progesterone levels were >1.8 ng/ml, although this declining has not clinical significance. In conclusion, clinical outcomes are not affected in high responders; moreover, the ROC curve values failed to find any prediction suggesting that the clinical consequences would be barely significant.

## Abbreviations

ANOVA: Analysis of variance; AUC: Area under the curve; E2: Estradiol; FET: Frozen-embryo transfer; FSH: Follicle stimulating hormone; GnRH: Gonadotropin-releasing hormone; hCG: Human chorionic gonadotropin; HP-hMG: Highly purified human menopausal gonadotropin; LH: Luteinizing hormone; OHSS: Ovarian hyperstimulation syndrome; OR: Odds ratio; P4: Progesterone; ROC: Receiver operating characteristics.

## Competing interest

The authors declare that they have no competing interests.

## Author’s contributions

AR played a role in conception and design, analysis and interpretation of the data, drafting the manuscript and revising it critically for important intellectual content, and final approval of the manuscript. MC was involved in drafting the manuscript. EB was involved in analysis and interpretation of the data and revising the manuscript for content. MM took part in data analysis and interpretation. JGV was involved in drafting the manuscript, providing important intellectual content and providing final approval. All authors read and approved the final manuscript.
